# Prognostic markers for the clinical course in the blood of patients with SARS-CoV-2 infection

**DOI:** 10.1186/s40001-022-00864-z

**Published:** 2022-11-21

**Authors:** Johannes C. Fischer, Vera Balz, Danny Jazmati, Edwin Bölke, Noemi F. Freise, Verena Keitel, Torsten Feldt, Björn-Erik Ole Jensen, Johannes Bode, Tom Lüdde, Dieter Häussinger, Ortwin Adams, E. Marion Schneider, Jürgen Enczmann, Jutta M. Rox, Derik Hermsen, Karin Schulze-Bosse, Detlef Kindgen-Milles, Wolfram Trudo Knoefel, Martijn van Griensven, Jan Haussmann, Balint Tamaskovics, Christian Plettenberg, Kathrin Scheckenbach, Stefanie Corradini, Alessia Pedoto, Kitti Maas, Livia Schmidt, Olaf Grebe, Irene Esposito, Anja Ehrhardt, Matthias Peiper, Bettina Alexandra Buhren, Christian Calles, Andreas Stöhr, Peter Arne Gerber, Artur Lichtenberg, Hubert Schelzig, Yechan Flaig, Amir Rezazadeh, Wilfried Budach, Christiane Matuschek

**Affiliations:** 1 Institute for Transplant Diagnostics and Cell Therapeutics, Medical Faculty, University Hospital Dusseldorf, Heinrich-Heine-University Dusseldorf, Dusseldorf, Germany; 2 Department of Radiation Oncology, Medical Faculty, University Hospital Dusseldorf, Heinrich-Heine-University Dusseldorf, Dusseldorf, Germany; 3 Department of Gastroenterology, Hepatology and Infectious Diseases, Medical Faculty, University Hospital Dusseldorf, Heinrich-Heine-University Dusseldorf, Dusseldorf, Germany; 4 Institute for Virology, Medical Faculty, University Hospital Dusseldorf, Heinrich-Heine-University Dusseldorf, Dusseldorf, Germany; 5 Division of Experimental Anesthesiology, University Hospital Ulm, Ulm, Germany; 6 Central Institute for Laboratory Diagnostics and Clinical Chemistry, Medical Faculty, University Hospital Dusseldorf, Heinrich-Heine-University Dusseldorf, Dusseldorf, Germany; 7 Department of Anesthesiology, Medical Faculty, University Hospital Dusseldorf, Heinrich-Heine-University Dusseldorf, Dusseldorf, Germany; 8 Department of Surgery and Interdisciplinary Surgical Intensive Care Unit, Medical Faculty, University Hospital Dusseldorf, Heinrich-Heine-University Dusseldorf, Dusseldorf, Germany; 9 Department cBITE, MERLN Institute for Technology-Inspired Regenerative Medicine, Maastricht University, Maastricht, The Netherlands; 10 Department of Ear, Nose and Throat Disease, Medical Faculty, University Hospital Dusseldorf, Heinrich-Heine-University Dusseldorf, Dusseldorf, Germany; 11 Department of Radiation Oncology, LMU University of Munich, Munich, Germany; 12 Department of Anesthesiology, Memorial Sloan Kettering Cancer Center, New York, NY USA; 13 Department of Cardiology and Rhythmology, Petrus Hospital, Wuppertal, Germany; 14 Institute of Pathology, Medical Faculty, University Hospital Dusseldorf, Heinrich-Heine-University Dusseldorf, Dusseldorf, Germany; 15 Institute of Virology, University of Witten/Herdecke, Witten, Germany; 16 Medical Faculty, Medical Faculty, University Hospital Dusseldorf, Heinrich-Heine-University Dusseldorf, Dusseldorf, Germany; 17 Coordination Center for Clinical Studies, Medical Faculty, University Hospital Dusseldorf, Heinrich-Heine-University Dusseldorf, Dusseldorf, Germany; 18 Department of Cardiac Surgery, Medical Faculty, University Hospital Dusseldorf, Heinrich-Heine-University Dusseldorf, Dusseldorf, Germany; 19 Department of Vascular Surgery, Medical Faculty, University Hospital Dusseldorf, Heinrich-Heine-University Dusseldorf, Dusseldorf, Germany

**Keywords:** SARS-CoV-2, Neutralizing antibodies, Persistence, HLA haplotypes, Chemokine receptor, CCR5, COVID-19, Clinical course of the disease

## Abstract

**Background:**

The presentation of peptides and the subsequent immune response depend on the MHC characteristics and influence the specificity of the immune response. Several studies have found an association between HLA variants and differential COVID-19 outcomes and have shown that HLA genotypes are associated with differential immune responses against SARS-CoV-2, particularly in severely ill patients. Information, whether HLA haplotypes are associated with the severity or length of the disease in moderately diseased individuals is absent.

**Methods:**

Next-generation sequencing-based HLA typing was performed in 303 female and 231 male non-hospitalized North Rhine Westphalian patients infected with SARS-CoV2 during the first and second wave. For HLA-Class I, we obtained results from 528 patients, and for HLA-Class II from 531. In those patients, who became ill between March 2020 and January 2021, the 22 most common HLA-Class I (HLA-A, -B, -C) or HLA-Class II (HLA –DRB1/3/4, -DQA1, -DQB1) haplotypes were determined. The identified HLA haplotypes as well as the presence of a CCR5Δ32 mutation and number of O and A blood group alleles were associated to disease severity and duration of the disease.

**Results:**

The influence of the HLA haplotypes on disease severity and duration was more pronounced than the influence of age, sex, or ABO blood group. These associations were sex dependent. The presence of mutated CCR5 resulted in a longer recovery period in males.

**Conclusion:**

The existence of certain HLA haplotypes is associated with more severe disease.

**Supplementary Information:**

The online version contains supplementary material available at 10.1186/s40001-022-00864-z.

## Introduction

Since the outbreak of the SARS-CoV-2 pandemic in late 2019, knowledge of the disease and its pathophysiology has rapidly evolved, leading to more effective therapeutic and prophylactic approaches, including vaccination [1, 2]. As a result of the global vaccination campaign, mortality and hospitalization due to COVID-19 have been significantly reduced, especially in developed countries [1–6]. However, the relative contribution of T-cell sensitization and neutralizing antibodies is still insufficiently understood [7]. SARS-CoV-2 continuously mutates and poses a significant challenge to cellular and humoral immunity and vaccination [1, 6]. Therefore, a better understanding of the immunological background of the disease is of utmost importance to access more effective prevention and treatment strategies.

SARS-CoV-2 infection is clinically heterogeneous, ranging from subclinical courses to states of critical disease stages with high mortality [2]. Systemic and mucosal immunity requires recognition of peptides bound to the MHC of antigen-presenting cells and subsequent T-cell activation and effector function. Identification of MHC restriction requirements could lead to better-personalized therapeutic strategies, especially when facing numerous vaccination failures [7].

It is reasonable to assume that the course of the disease might depend on the adaptive patients' immune response. In addition, innate immunity by, e.g., less polymorphic TCR gamma/delta T cells, which are mainly active in mucosal tissues, might play a role [8].

Through its essential role in distinguishing between endogenous and exogenous cells [9, 10], the genetic polymorphisms of Human Leukocyte Antigen (HLA) alleles appear to influence the clinical course of patients infected with various RNA viruses, resulting in either protection or enhanced viral persistence [11]. Several HLA polymorphisms have been identified as individual genetic characteristics that affect the individual immune response towards SARS-CoV-2 as well as virus persistence and a predisposition to develop autoimmune diseases [12]. Conflicting results have been published regarding the role of the ABO blood group system, prompting further investigation [13, 14]. Amoroso et al. found that HLA antigens could influence SARS-CoV-2 infection and clinical evolution of COVID-19. They endorsed that blood group A individuals are at greater risk of infection and provided indications on the spread of the disease and clues about infection prognosis and vaccination strategies [9]. Pisanti et al. found that the two most frequent HLA haplotypes in the Italian population, HLA-A*01:01G-B*08:01G-C*07:01G-DRB1*03:01G and HLA-A*02:01G-B*18:01G-C*07:01G-DRB1*11:04G, had a regional distribution overlapping that of COVID-19 and showed, respectively, a positive (suggestive of susceptibility) and negative (suggestive of protection) significant correlation with both COVID-19 incidence and mortality [15]. Other factors that appear to model the individual response include the chemokine receptor CCR5, which plays a role in the humoral response of mucosal immunity to various infections [16–18].

Having access to a clinically and predominantly Caucasian patient population, we examined the influence of the most common HLA haplotypes and genotypes, ABO blood group antigens, and the CCR5Δ32 mutant of CD195 concerning to their role in the clinical course. In addition, we compared the results for males and females since differences in severity and mortality are known for SARS-CoV 2 infection [19] and differences for disease susceptibility are discussed [19]. In the context of the pandemic, it is essential to understand the sensitivity of T cells among different population groups due to the differences in their HLA haplotypes. Characterization of immune response among population groups can help to design personalized therapies for patients with high disease severity. Our study analyzed a healthy population (of potential plasma donors) infected with SARS-CoV-2.

## Patients, materials and methods

### Patients

A total of *n* = 541 donors were recruited who were willing to donate convalescent plasma after confirmed SARS-CoV2 infection (between January 2020 and January 2021) were subsequently tested for their HLA-Class I (*n* = 528) and -Class II genotypes (*n* = 531), their ABO blood groups, and the presence or absence of the CCR5Δ32 mutant (rs6795991, *n* = 534) for prognostic clinical significance. Disease duration severity was assessed by a questionnaire. Severity was graded according to the WHO COVID-scale [20] into mild disease (WHO° I and IIa) and moderate disease (WHO° IIb and III). All plasma donors were followed up clinically for 600 days. Written informed consent was obtained from all participants for the study. The ethics committee approved the study of Heinrich-Heine-University Dusseldorf, Germany.

All experimental studies were performed according to manufacturers’ standard operating procedures and international guidelines.

### Antibody determination

Antibody determination was performed using two serological assays as described before [10, 21]. Spike S1 protein domain specific IgA and IgG antibodies were detected by ELISA (Euroimmun, Germany), Ig antibodies against the Nucleocapsid were verified by Roche Diagnostic Test (Elecsys^®^).

In addition, neutralizing antibodies were tested. All assays were performed in two samples. Donor plasma was heat inactivated and diluted. Serum neutralization titer was determined by microscopic inspection as the highest serum dilution without virus-induced cytopathic effect (CPE). As positive controls, two previously tested sera from SARS-CoV-2 infected individuals were tested simultaneously in each round. A high-titer control (NT 1:640) and a medium-titer control (NT 1:160) were defined to validate each assay. Inter-assay and intra-assay variations were determined, and these control sera exhibited maximum variation within only one dilution level. In addition, serum from individuals not infected with SARS-CoV-2 and cells without serum were used as negative controls to confirm virus-induced CPE. A neutralization titer of 1:160 was defined as the threshold titer for the binary representation of virus neutralization capacity, allowing the potential plasmapheresis product to serve as a therapeutic agent.

### Genotyping and haplotype phasing

To genotype for HLA, CCR5 wild type (wt) and CCR5Δ32 mutant as well as for ABO blood group alleles, an amplicon-based NGS-based approach was used. For HLA six multiplex polymerase chain reactions (PCRs) were used to amplify exons 2, 3 and 4 of the HLA-Class I and HLA-DPB1 genes, exons 2 and 3 of HLA-DRB1, -DQA1 and -DQB1, and exon 3 of the CCR5 gene. The resulting fragments were supplemented with sample-specific barcodes and Illumina-compatible adapters. Sequencing was performed on an Illumina MiSeq device (Illumina Inc., San Diego, USA). A customized software (NGSSequence Analyser, Institute for Transplantation Diagnostics and Cell Therapy, University Hospital Dusseldorf, Dusseldorf, Germany) served for the analysis of the sequence reads using quality control values and high coverage to automate the data analysis. For haplotype phasing of the obtained HLA data, we used the Arlequin software (version 3.5.2.2, available cmpg.unibe.ch/software/arlequin35/), for binding affinities to viral peptides https://t-cov.hse.ru[22].

### Statistics

Statistical inference between two groups of interest was assessed by t-test statistics. Kaplan–Meier curves and two-sided log rank tests were used to evaluate the duration-of-infection times between groups of interest. If necessary (violation of the assumption of probability), Bonferroni correction was applied to counteract the multiple comparisons problem. Multivariable Cox regression analysis was performed to identify significant predictors for duration-of-infection, logistic regression for predictors for severity of the disease. Effects of the multivariate Cox regression models were measured using hazard ratio estimates with corresponding 95% confidence intervals, equivalent for the odds ratio in logistic regression. The distribution of the investigated alleles as well as the found haplotypes were tested for their hetero- and homozygosity by checking, whether they fulfill the conditions for a proving if the Hardy–Weinberg equilibrium (available https://ihg.helmholtz-muenchen.de/ihg/snps.html). Only haplotypes that were present in the population at least 5 times were considered. All p-values were determined using two-sided tests, and p-values of less than 0.05 were considered statistically significant. Analyses were performed using IBM SPSS Statistics for Windows (Version 22.0. Released 2013, Armonk, NY: IBM Corp) and R/ R-based blueskystatistics (version 3.6.3, https://www.r-project.org/, version 7.5, https://www.blueskystatistics.com).

## Results

A total of 541 plasma donors (median age 43.9 ± 12.5 years), 231 males (median age 43.5 ± 13.0 years) and 310 females (median age 43.5 ± 12.2 years) with mild (*n* = 320) and moderate disease (*n* = 221) were included in this analysis. All plasma donors were healthy with an ECOG status of 0 prior infection with SARS-CoV-2, e.g., no co-morbidities like infectious diseases, malignant tumors or immunocompromised. The duration of the disease was 15 ± 7 days. Our patients with SARS-CoV-2 were never hospitalized, and symptoms corresponded to WHO° 1–3 [20]. During the first wave of infection with SARS-CoV-2, we investigated 179 patients (91 males, 88 females), and during the second wave of infection, we studied 362 convalescent plasma donors (140 males, 222 females). The characteristics of all investigated donors are shown in Table 1. In total, a correlation was found between the severity of the clinical picture and the duration of the disease course. In total, patients of wave 1 showed less mild course than patients of wave 2 (*p* < 0.003). Patients of the 2nd trends to have a shorter course of disease (n.s. *p* < 0.1). The more pronounced the patient's symptoms, the longer their clinical course and the higher the reached antibody levels (data not shown). We found elevated or stable period Ig N antibody levels of up to 240 days after the end of symptoms. In all patients in whom elevated antiviral IgG antibodies had been observed the respective anti-S IgG antibodies remained detectable for least 240 days as we reported previously for the patients of the first wave [10, 23].

**Table 1:** Characteristics of all investigated patients

Characteristics of all patients		
	Male	Female
Number	*N* = 231	*N* = 310
Age	43.5 ± 13.0 (18.3-77 years)	43. 5 ± 12.2 (18.7–76.1 years)
Mild diseasee WHO 1-2A	128	192
Moderate disease WHO 2B-3	103	118
Severe disease WHO > 3	0	0
Mortality	0	0
ECOG performance status prior SARS-CoV-2 infection	0	0

Characteristics of patients infected during the 1st wave with SARS-CoV-2 (25th January 2020 until 16th May 2020)		
	Male	Female
Number	*N* = 91	*N* = 88
Age	42.9 ± 13.3 (19.7–76.8 years)	45.3 ± 13.7 (19.9–79.5 years)
Mild disease WHO 1-2A	39	47
Moderate disease WHO 2B-3	52	41
Severe disease WHO > 3	0	0
Mortality	0	0
ECOG performance status prior SARS-CoV-2 infection	0	0

Characteristics of patients infected during the 2nd wave with SARS-CoV-2 (19th May 2020 until 30th January 2021)		
	Male	Female
Number	*N* = 140	*N* = 222
Age	43.3 ± 13.2(18.3-77 years)	43.5 + 13.5 (18.7–79.5 years)
Mild disease WHO 1-2A	89	145
Moderate disease WHO 2B-3	51	77
Severe disease WHO > 3	0	0
Mortality	0	0
ECOG performance status prior SARS-CoV-2 infection	0	0

### Association of HLA haplotypes and severity of COVID-19

ABO blood group (BG) genotype could be assessed in 532 patients, for O alleles in 532 cases, for A alleles in 532 cases, for CCR Δ32 in 534 cases.

Haplotype phasing of the obtained HLA data revealed several HLA-Class I and Class II haplotypes (Table 2). For HLA-Class I, we obtained results from 528 patients, and for HLA-Class II from 531.

**Table 2:** The frequencies of the examined HLA haplotypes

Haplotype	Abbreviation	No	Yes (heterozygote)	Yes (homozygous)
HLA-Class I (*n* = 528)				
A*01:01_B*08:01_C*07:01	HLA_ABC_01	454	73	1
A*03:01_B*07:02_C*07:02	HLA_ABC_02	465	60	3
A*02:01_B*44:02_C*05:01	HLA_ABC_03	471	56	1
A*02:01_B*07:02_C*07:02	HLA_ABC_04	493	35	0
A*02:01_B*40:01_C*03:04	HLA_ABC_05	505	20	3
A*02:01_B*13:02_C*06:02	HLA_ABC_06	511	17	0
A*24:02_B*07:02_C*07:02	HLA_ABC_07	511	16	1
A*02:01_B*35:01_C*04:01	HLA_ABC_08	514	14	0
A*01:01_B*35:01_C*04:01	HLA_ABC_09	516	12	0
A*02:01_B*15:01_C*03:03	HLA_ABC_10	513	15	0
A*03:01_B*35:01_C*04:01	HLA_ABC_11	516	12	0
A*01:01_B*57:01_C*06:02	HLA_ABC_12	519	9	0
A*02:01_B*15:01_C*03:04	HLA_ABC_13	518	10	0
A*11:01_B*35:01_C*04:01	HLA_ABC_14	518	10	0
A*02:01_B*08:01_C*07:01	HLA_ABC_15	518	10	0
A*02:01_B*51:01_C*15:02	HLA_ABC_16	523	5	0
A*01:01_B*07:02_C*07:02	HLA_ABC_17	519	9	0
A*02:01_B*18:01_C*07:01	HLA_ABC_18	518	10	0
A*02:01_B*27:05_C*02:02	HLA_ABC_19	515	13	0
A*02:01_B*57:01_C*06:02	HLA_ABC_20	516	12	0
A*03:01_B*40:01_C*03:04	HLA_ABC_21	519	9	0
A*29:02_B*44:03_C*16:01	HLA_ABC_22	520	8	0
HLA-Class II (*n* = 531)				
DRB1*07:01_DQA1*02:01_DQB1*02:01	HLA_DRB_DQA_DQB_01	436	89	6
DRB1*15:01_DQA1*01:02_DQB1*06:02	HLA_DRB_DQA_DQB_02	401	126	4
DRB1*01:01_DQA1*01:01_DQB1*05:01	HLA_DRB_DQA_DQB_03	427	99	5
DRB1*03:01_DQA1*05:01_DQB1*02:01	HLA_DRB_DQA_DQB_04	427	97	7
DRB1*11:01_DQA1*05:01_DQB1*03:01	HLA_DRB_DQA_DQB_05	455	70	6
DRB1*07:01_DQA1*02:01_DQB1*03:01	HLA_DRB_DQA_DQB_06	495	36	0
DRB1*13:01_DQA1*01:03_DQB1*06:03	HLA_DRB_DQA_DQB_07	462	66	3
DRB1*04:01_DQA1*03:01_DQB1*03:02	HLA_DRB_DQA_DQB_08	481	49	1
DRB1*11:04_DQA1*05:01_DQB1*03:01	HLA_DRB_DQA_DQB_09	484	45	2
DRB1*04:01_DQA1*03:01_DQB1*03:02	HLA_DRB_DQA_DQB_10	492	39	0
DRB1*08:01_DQA1*04:01_DQB1*04:02	HLA_DRB_DQA_DQB_11	496	35	0
DRB1*13:02_DQA1*01:02_DQB1*06:04	HLA_DRB_DQA_DQB_12	504	27	0
DRB1*14:54_DQA1*01:04_DQB1*05:03	HLA_DRB_DQA_DQB_13	505	25	1
DRB1*04:04_DQA1*03:01_DQB1*03:02	HLA_DRB_DQA_DQB_14	512	18	1
DRB1*12:01_DQA1*05:01_DQB1*03:01	HLA_DRB_DQA_DQB_15	512	19	0
DRB1*13:03_DQA1*05:01_DQB1*03:01	HLA_DRB_DQA_DQB_16	513	18	0
DRB1*16:01_DQA1*01:02_DQB1*05:02	HLA_DRB_DQA_DQB_17	516	15	0
DRB1*09:01_DQA1*03:02_DQB1*03:03	HLA_DRB_DQA_DQB_18	519	12	0
DRB1*04:07_DQA1*03:02_DQB1*03:01	HLA_DRB_DQA_DQB_19	521	10	0
DRB1*01:02_DQA1*01:01_DQB1*05:01	HLA_DRB_DQA_DQB_20	526	5	0
DRB1*11:03_DQA1*05:01_DQB1*03:01	HLA_DRB_DQA_DQB_21	523	8	0
DRB1*10:01_DQA1*01:04_DQB1*05:01	HLA_DRB_DQA_DQB_22	526	5	0

Frequencies of homozygous and heterozygous variant carriers were tested for each allele/haplotype, whether they fulfill the assumption of the Hardy–Weinberg equilibrium (HWE) in the whole group as well as in the mild and moderate disease group at a significance level of less than *p* < 0.02 (see Additional file 1). Deviations from the HWE (DHWE) were found for number of OBG alleles with more homozygous O-carriers, based on more carriers in the mild disease group. DHWE was also seen for the HLA haplotype A*02:01_B*40:01_C*03:04 (HLA-ABC_05), due to more homozygous than expected, In haplotype HLA-DRB1*03:01_DQA1*05:01_DQB1*02:01 (DRB-DQA_DQB_4) 6 out of 7 homozygous were seen for in the moderate diseased group (DHWE), whereas all of the 6 homozygous carriers of the HLA haplotype DRB1*11:01_DQA1*05:01_DQB1*03:01 (DRB_DQA_DQB_05) were found in the mild disease group (DHWE).

Univariate analysis of the HLA haplotypes showed that the presence the HLA-Class II haplotype DRB1*07:01_DQA1*02:01_DQB1*03:01 (36/531 6.8%, OR 2.16, 95% confidence interval 1.09–4.36, “DRB_DQA_DQB_06”) was predictive for a mild disease course of SARS-CoV-2 infection. Two haplotypes (class I A*02:01_B*07:02_C*07:02 (35/528, 6.6%, OR 0.28 95% CI 0.10–0.65, “ABC_04”); class II DRB1*12:01_DQA1*05:01_DQB1*03:01 (19/531, 2,8%, OR 0.164 95%CI 0.026–0.58, “DRB_DQA_DQB_15”)) were associated with a more severe disease course.

**Fig. 1:**
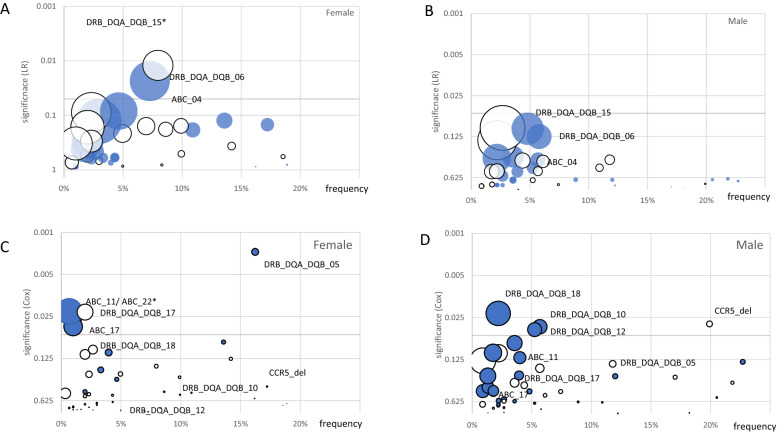
Univariate association of HLA haplotypes with mild/moderate disease (relative risk, OR, log rank (LR)) in females (**A**) and males (**B**) and for disease duration (hazard ratio, (Cox)) in females (**C**) and males (**D**). Haplotype frequency within the group is plotted on the x-axis and the y-axis shows the significance levels of the OR for moderate disease / HR for disease duration. The size of the spheres indicates the effect size and was calculated as absolute value of the logarithms of OR/HR. Full spheres represent a favorable association with OR or HR, and empty spheres represent an association with a higher RR or HR. The 0.05 significance level is shown as parallel to the x-axis. Alleles/haplotypes which were either significant in females or males for OR (panel **A** and **B**) are denoted in both panels. Same applies for HR (panel **C** and **D**). *Note that the sphere for the HLA-Class II haplotype DRB_DQA_DQB_15 in panel A is not on scale, since all females homozygous for this haplotype had had a mild course of disease (*n *= 8)

When looking for males and females separately, we found only in females a significant association for the presence of haplotype “ABC_04” and “ DRB_DQA_DQB_06” with severity of the disease (Fig. 1a, b).

In the whole group the HLA-Class I haplotype A*03:01_B*35:01_C*04:01 (12/528, 2,3%, HR 2.1 95%CI 1.19–3.75, “ABC_11”) was associated with shorter disease, the HLA-Class II haplotype DRB1*15:01_DQA1*01:02_DQB1*06:02 (126/531, 25,5% OR 0.81 95%CI 0.66–0.99, “DRB_DQA_DQB_05”) with longer duration. Looking at females only, HLA-Class I haplotype “ABC_11” (A*03:01_B*35:01_C*04:01, HR 3.8 95%CI 1.2–11.8), haplotype ABC_17 (A*01:01_B*07:02_C*07:02, HR 3.4, 95%CI 1.1–10.6), and ABC_22 (A*29:02_B*44:03_C*16:01 HR 5.30 95%CI 1.29–2.73) with shorter duration as well as HLA-Class II haplotype DRB_DQA_DQB_05 (HR 1.6 CI 1.2—2.2). HLA-Class II haplotype DRB_DQA_DQB_17 was associated with longer duration (DRB1*16:01_DQA1*01:02_DQB1*05:02, HR 0.34 95%CI 0.14–0.85) (Fig. 1c).

In males also HLA-Class II haplotypes reached significance DRB_DQA_DQB_10 (DRB1*04:01_DQA1*03:01_DQB1*03:01; HR 1.88 95%CI 1.04–3.40), DRB_DQA_DQB_12 (DRB1*13:02_DQA1*01:02_DQB1*06:04; HR 1.85 95%CI 1.03–3.34), HLA_DRB_DQA_DQB_18 (DRB1*09:01_DQA1*03:01_DQB1*03:03; HR 2.86 95%CI 1.17–6.99) for shortening the disease (Fig. 1d).

Within multivariate analysis for severity of disease, beside age, gender, CCR5Δ32 alleles, number of blood group O alleles all HLA haplotypes were included, which showed either a Hardy Weinberg deviation and/or a univariate significance for association with severity of the disease in males and/ or females. Analysis was further weighted for wave of infection.

**Fig. 2:**
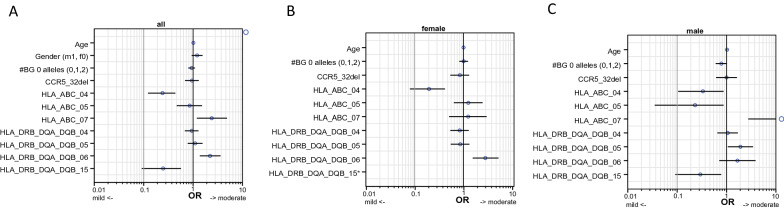
Multivariate association of age, gender, and HLA haplotypes as risk factors for disease severity (WHO° I & IIa (mild) vs WHO° IIb & III). **A** shows results for the whole group, **B** for females, **C** for males.*Note that for the HLA-Class II haplotype DRB_DQA_DQB_15 in panel B whisker is not on scale, since all females homozygous for this haplotype had had a mild course of disease (*n* = 8)

Results for the whole group are shown in Fig. 2a, for females in Fig. 2b and for males in Fig. 2c. Most haplotypes showed the same association in favoring mild or moderate disease: HLA haplotype ABC_04 (A*02:01_B*07:02_C*07:02) favors mild disease in the whole group (*p* < 0.001) as well in females (*p* < 0.001) and males (*p* < 0.04), ABC_07 (A*24:02_B*07:02_C*07:02) in the whole group (*p* < 0.015), males (*p* < 0.004), in females n.s. but also favoring in direction more moderate disease.

DRB_DQA_DQB_06 (DQA1*02:01_DQB1*03:01) in the whole group *p* < 0.002, females (*p* < 0.001), in males n.s. but also favoring more moderate disease.

DRB_DQA_DQB_15 (DRB1*12:01_DQA1*05:01_DQB1*03:01) in the whole group (*p* < 0.003), in males (p < 0.025) favoring mild disease. All 8 females carrying this haplotype had also mild disease.

Haplotype ABC_05 (A*02:01_B*40:01_C*03:04) favors only in males for mild disease (*p* < 0.05).

DRB_DQA_DQB_05 (DRB1*11:01_DQA1*05:01_DQB1*03:01) favors for more moderate disease only in males.

**Fig. 3:**
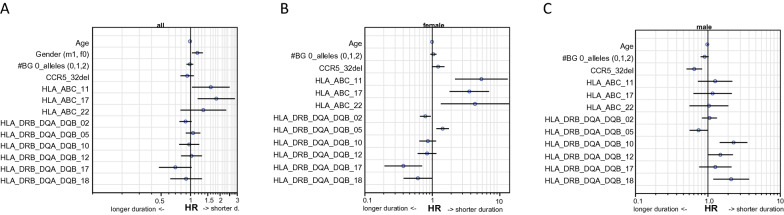
Multivariate association of age, gender, and HLA haplotypes as risk factors for disease duration (longer disease (HR < 1) vs. shorter disease duration (HR > 1). **A** shows results for the whole group, **B** for females, **C** for males

Multivariate analysis for duration of disease included age, gender, CCR5Δ32 alleles, number of blood group O alleles and all those HLA haplotypes, which showed a univariate significance for association duration of the disease in males and/ or females (Fig. 3a).

Age was associated with a significant, but small impact to prolong the disease (*p* < 0.001, HR 0.987), males had a shorter illness course (HR 1.17, *p* < 0.015). CCR5Δ32 mutation only impacted males (HR 0.65, *p* < 0.001) but not females.

Following HLA-Class I haplotypes were associated with shorter disease duration: HLA_ABC_11 (A*11:01_B*35:01_C*04:01; whole group HR 1.61, *p* < 0.035, in females HR 5.33, *p* < 0.001, in males n.s. but same direction of influence), HLA_ABC_17 (A*01:01_B*07:02_C*07:02, whole group HR 1.83, *p* < 0.007, in females HR 3.53, *p* < 0.001, in males n.s. but same direction of influence). In females in addition haplotype HLA_ABC_22 (A*29:02_B*44:03_C*16:01; HR 1.45, *p* < 0.015) showed same association.

In men, HLA_DRQ_DQA_DQB_10 (DRB1*04:01_DQA1*03:01_DQB1*03:02, HR 2.26, *p* < 0.001) was also associated with shorter disease course.

Haplotype HLA_DRQ_DQA_DQB_17 (DRB1*16:01_DQA1*01:02_DQB1*05:02) was associated with longer disease course in females (HR 0.37, *p* < 0.003), as was HLA_DRQ_DQA_DQB_02 (DRB1*15:01_DQA1*01:02_DQB1*06:02, HR 0.79, *p* < 0.02).

Two haplotypes were associated with an opposite effect in males and females: HLA_DRQ_DQA_DQB_05 (DRB1*11:01_DQA1*05:01_DQB1*03:01, HR_females_ 1.42 (supporting shorter disease) *p*_females_ < 0.002; HR_males_ 0.74 *p*_males_ < 0.043) and, vice versa HLA_DRQ_DQA-DQB_18 (DRB1*09:01_DQA1*03:02_DQB1*03:03, HR_females_ 0.61 *p*_females_ < 0.05; HR_males_ 2.26 (supporting shorter disesease) *p*_males_ < 0.001).

**Fig. 4:**
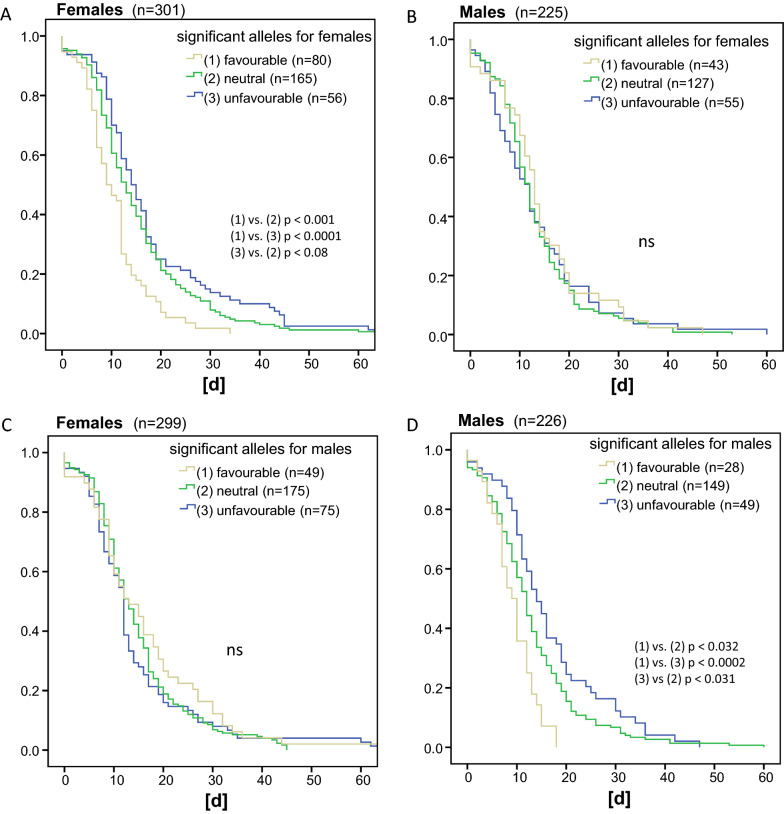
Kaplan–Meier estimates for the impact of disease specific favorable or unfavorable HLA haplotypes on duration of the disease when coded for significant alleles in females (**A**&**C**) or males (**B**&**D**)

When coding the beneficial and the disadvantageous HLA haplotypes for disease duration specific for sex separately (Fig. 4), females carrying beneficial female haplotypes had a median disease duration of 10 days (men 12 days), women carrying unfavorable haplotypes had a disease duration of 14 days (men 13 days). Women carrying either favorable and unfavorable or neither of those haplotypes, had a median disease duration of 12 days.

When looking at the male specific haplotypes, males carrying beneficial male haplotypes had median disease duration of 10 days (females 12 days), men carrying unfavorable haplotypes 14 days (females 13 days), men with unfavorable and favorable or neither of those haplotypes had a disease duration of 12 days (females 13 days). For significance levels, see Fig. 4.

**Fig. 5:**
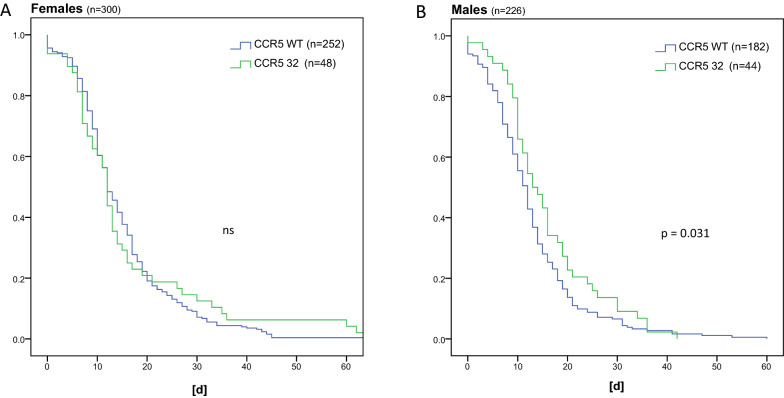
Kaplan–Meier estimates for the impact of CCR5 wt and CCR5Δ32 on disease duration in females (**A**) and males (**B**)

We further analyzed the clinical outcome of plasma donors specifically for the heterozygous CCR5Δ32 mutation. We investigated whether this mutation had an impact on the duration of symptoms. In patients infected during the 1^st^ wave (up until week 20/2020), heterozygous mutation of CCR5Δ32 was associated with a significantly longer disease course (*p* = 0.013) [10]. This significant difference was confirmed for the whole group only in males (Fig. 5, *p* < 0.032).

The influence of the HLA haplotypes on disease severity and duration was stronger than the influence of age, sex, ABO blood group, or wave of infection with different mutants of the virus.

Numbers of in silico predicted viral T-cell epitopes did not correlate with the severity of SARS-CoV-2 infections.

## Discussion

Since the onset of the SARS-CoV-2 pandemic, the heterogeneity of the clinical course has been an essential issue for investigations. While in some patients, the infection goes unnoticed, in others, the same infective agent leads to a severe course of disease accompanied by high morbidity and mortality [21, 24]. Besides the immune reaction of the patient itself, there could be a combination of several factors that lead to these different outcomes like gender, distance regulations and length of the infection wave or season.

Nevertheless, individual immunologic pathways based on metabolism, age, sex and genetics are known to play a crucial role [25]. Our study demonstrates a prolonged or more severe clinical course in carriers of some HLA haplotypes while other haplotypes favor shorter or milder outcomes.

Carriers of the HLA haplotypes HLA-A*02:01_B*07:02_C*07:02 and HLA-DRB1*12:01_DQA1*05:01_DQB1*03:01 had only mild disease symptoms, whereas carriers of the haplotypes HLA-A*24:02_B*07:02_C*07:02 and HLA-DRB1*07:01_DQA1*02:01_DQB1*03:03 and (especially in men) HLA-DRB1*11:01_DQA1*05:01_DQB1*03:01 showed more moderate/severe symptoms.

As with others [26], we found no strong correlation with previously described class I superhaplotypes [27], although patients with haplotypes containing the A24 superhaplotype motif tend to have a more unfortunately course, whereas patients bearing the B07/B44 motif without A24 trend to have a favorable course. Regarding class II superhaplotypes [28], patients with DR1/DQ1 and DR3/DQ3 superhaplotypes is among those doing worse, whereas beneficial haplotypes contain the superhaplotypes DR4, DR5 and DR9.

SARS-CoV-2 infection and its severity might be related to gender, and males are more likely to develop COVID-19 [29]. Although males are more susceptible to most viral infections, females possess immunological features that render them more vulnerable to distinct immune-related disease outcomes [30]. Interestingly, in our cohort females reported a longer disease duration, whereas men tend to report more severe disease. We questioned whether investigated alleles and or haplotypes may have a differential impact on severity and duration within this non-hospitalized patient cohort. When looking at the severity of the disease, the pattern of haplotypes being favorable or unfavorable differ not relevantly between the genders. When looking at disease duration, which is also a sign of disease control, interestingly three class I haplotypes are associated with shorter disease duration in females, whereas in men class II haplotypes are positively associated with shorter disease—in line with observations, that a defined antigen stimulus would lead to more clonal expansions in CD8 T cells in men than in women [31] and thus differences in class I repertoire may impact disease control in women more than in men. On the other side, women show per se higher T-helper cell levels and activability [32], which leads to the conclusion that differences within the HLA-Class II repertoire on disease control may only be seen in men.

As known, the C-C chemokine receptor 5 (CCR5) is responsible for immune and inflammatory responses by mediation of chemotactic activity in T cells, although it is expressed on different cell types. It has been shown to act as co-receptor for the human immunodeficiency viruses (HIV-1, HIV-2), resulting in resistance to HIV infection in homozygous CCR5Δ32 mutation carriers. CCR5 serves not only as a co-receptor, but also contributes to the functional fitness of CD4 + T cells themselves. CCR5 signaling reduces ceramide levels and thereby increases HLA experienced T‐cell antigen receptor (TCR) nanoclustering in antigen‐experienced CD4 + T cells [7]. This contributes to the more severe outcome in, e.g., early summer meningoencephalitis or West Nile fever in CCR5Δ32 carriers [33]. Previously, in patients of the 1st wave, we noticed that CCR5Δ32 carriers had a longer disease course [10]. If we look now at the not so severely ill patients of the 2nd wave, we observe this only in males. This corresponds to two expectations: first, in the not so severely ill population this effect can only be seen in the group where an influence of the MHC class II repertoire is seen for duration of the disease, as we see in males and, second, due to the association of female gender with increased CD4 + T cell CCR1–CCR5 gene and protein expression [34], the increased CCR expression, which correlates with enhanced in vitro chemotaxis response, the influence of the mutation will be reversed. As others in larger cohorts, we saw no impact of the CCR5Δ32 mutation on the primary severity of the disease [17]. In our cohort, the prevalence of the mutation was 17.4%, but 11.5% in the 26 asymptomatic patients. In a larger cohort of 164 asymptomatic SARS-CoV-2 infected patients Hubacek et al. noticed that during the 1st wave the incidence of CCR5Δ32 was higher in (23.8%) than in 252 symptomatic patients (16.7%) [18]. This results of a protective role of the mutation could not confirmed by the analysis in 110,544 stem cell donors [17]. When looking at critical ill patients, which were not included in our study, CCR5 might mediate immune dysregulation and thus leronlimab, a CCR5 blocking antibody may limit hyperinflammation in critical ill patients [16]. As of September 2022, randomized clinical trials evaluating leronlimab in patients with COVID-19 could not confirm this (NCT04343651) or are not recruiting (NCT04347239).

Previously, we found that ABO heterogeneity correlated with a higher risk of a more severe disease in patients of the 1st wave [10]. Although this effect is now no longer visible to the same extent, it is striking that for the blood group O allele the Hardy–Weinberg equilibrium is violated: a disproportionately large number of blood group O allele homozygotes are found in the group of those with only mild disease.

However, our study has several limitations. First, since it only covers patients of the 1st and 2nd wave before variants of concern emerged in Germany, the found associations are related to the original variant. Although we compared the patients of the first and second wave together, they differ: in the first group, most of the patients were skiers who came from a SARS-CoV-2 hotspots in Austria. In the 2nd group women predominated. They were recruited following public calls for convalescent plasma donation, where usually more women than men respond. It could be assumed, that in the first wave, which occurred in the cold season, patients were exposed to a higher viral load due to a lack of protective strategies such as clearance regimens or protective masks. In addition, the second wave occurred in the summer. Also, all patients were non-hospitalized – the highest WHO° was III. Finally, all patients were potentially able to donate blood and, therefore, primarily healthy individuals without severe co-morbidities before the infection.

Although limited by the size and retrospective nature of this cohort study, we were able to identify HLA haplotypes associated with the severity and duration of disease in 541 SARS-CoV-2 infected patients. While we found no sex association for disease severity and association with the haplotypes studied, we did see one for disease duration. Two of the 22 HLA-Class I haplotypes studied were significantly associated (HR > 2) with a shorter disease course in females. In contrast, two of the 22 HLA-Class II examined were favorably associated with disease duration only in men (HR > 2). Men with a CCR5Δ32 mutation had a longer disease course.

## Supplementary Information

**Additional file 1: Table S1.** Tests for deviation and tests for association from Hardy Weinberg equilibration.

## Data Availability

Data are applicable from JCF and CM.

## Abbreviations

CCR5: C–C chemokine receptor type 5
CD4: Cluster of differentiation 4
COVID-19: Coronavirus disease 2019
CP: Convalescent plasma
CPE: Cytopathic effect
e.g.: Exempli gratia
ELISA: Enzyme-linked immunosorbent assay
HIV: Human immunodeficiency virus
HR: Hazard ratio
HLA: Human leukocyte antigen system
Ig: Immunoglobulin
IL: Interleukin
IQR: Inter-quartile range
MCP: Monocyte chemo-attractant protein
MHC: Major histocompatibility complex
PCR: Polymerase chain reaction
RNA: Ribonucleic acid
SARS-CoV-2: Severe acute respiratory syndrome coronavirus 2
WHO: World Health Organization
Wt: Wild type
